# Color and Marbling as Predictors of Meat Quality Perception of Argentinian Consumers

**DOI:** 10.3390/foods10071465

**Published:** 2021-06-24

**Authors:** María Laura Testa, Gabriela Grigioni, Begoña Panea, Enrique Pavan

**Affiliations:** 1 Instituto Nacional de Tecnología Agropecuaria, Avenida Rivadavia 1439, Ciudad Autónoma de Buenos Aires C1033AAE, Argentina; testa.marialaura@inta.gob.ar (M.L.T.); grigioni.gabriela@inta.gob.ar (G.G.); 2 Consejo Nacional de Investigaciones Científicas y Técnicas CONICET, Godoy Cruz 2290, Ciudad Autónoma de Buenos Aires C1425FQB, Argentina; 3 Centro de Investigación y Tecnología Agroalimentaria de Aragón (CITA), Unidad de Producción y Sanidad Animal, Avenida de Montañana 930, 50059 Zaragoza, Spain; bpanea@cita-aragon.es; 4 Instituto Agroalimentario de Aragón—IA2 (CITA-Universidad de Zaragoza), Avda. Montañana 930, 50059 Zaragoza, Spain; 5 Facultad de Ciencias Agrarias, Universidad Nacional de Mar del Plata, Ruta Nacional 226 km 73.5, c.c. 276, Balcarce 7620, Argentina; 6 Department of Animal and Veterinary Sciences, Clemson University, Clemson, SC 29634, USA

**Keywords:** consumer preferences in Argentina, beef, color, marbling

## Abstract

In Argentina, color and intramuscular fat are the main attributes of raw beef quality; however, it is necessary to clarify how consumers use them, in order to establish different marketing strategies. Consumer preferences are affected by multiple factors. Thus, the objective of the present study was to identify the Argentinian consumer’s choice behavior regarding beef color and fat content. An online survey was carried out in Argentina. It inquired about socio-demographic characteristics, purchase and consumption habits and beliefs, showing pictures related to color, marbling and the amount of fat. Choice behavior was evaluated by asking why consumers chose a particular picture out of the ones shown. Several Kruskal–Wallis tests evaluated the different hypotheses. Three different decision trees using the CHAID analysis method were created. Multifactorial analysis was carried out for clustering consumers. Regarding consumer beliefs, 90% of the respondents agreed with the sentence, “The two main characteristics defining beef quality at purchase time are meat color and marbling”. Socio-demographic characteristics affected purchase habits and beliefs; they also affected perceptions about meat color and marbling. It was possible to build three consumer groups for future marketing strategies: “hedonic” focused on a pleasing sensory experience, “appearance” prioritized the visual aspects, and the “health-conscious” consumers were interested in their healthy nutrition.

## 1. Introduction

With a total livestock population of 53.9 million cattle, Argentina produces more than 3 million tons of meat per year, and it was the fourth-largest producer of beef meat in 2018 [1]. Argentina is also well-known in the world for its good-quality beef product, and it is one of the major world exporters of beef. In addition, it is the second country in the world by per capita consumption of meat, which is around 100 kg per person/year. In Argentina, when buying beef, consumers base their choice mainly on color and intramuscular fat [2]. According to a survey carried out by the Argentinian Beef Promotion Institute [3], color, tenderness and intramuscular fat are the main beef quality attributes. However, it remains unclear whether consumers perceive and use these intrinsic attributes in different ways, especially since, in recent years, consumers have become increasingly aware of the relationship between food and health. In this sense, Argentinian meat consumption exceeds the nutritional recommendations for the prevention of chronic non-communicable diseases and some types of cancer. In Argentina, public health policies recommend a healthier diet [4], reducing the total meat consumption and increasing vegetables, fruits, and whole grains [5]. These recommendations may have changed the consumer perception of meat. On the other hand, the beef production system changed during the last two decades, due to a dramatic expansion in crop-growing areas, driven by increasing grain prices. This might also have produced a change in consumer perception [6], as the traditional beef production system of Argentina, previously based on pasturing only, is now complemented by a feedlot finishing period (2–3 months), which produces meat with greater fat content.

Despite all this, Argentinian consumer preferences for meat are infrequently studied. The few perception surveys developed in Argentina have focused mainly on the urban population and, especially, that of Buenos Aires city [7,8,9,10]; but as far as we know, there is no survey that has been carried out across the entire country. However, according to Zapata et al. [11], there is a marked difference in the food consumption patterns between rural and urban households in Argentina. Moreover, the authors showed consumption as affected by multiple factors like availability, accessibility, and food choice, which in turn can be influenced by geographic location, demographic condition, income, socioeconomic level, globalization, commercialization, religion, culture and attitudes of consumers. For instance, meat perception by rural consumers may be determined by their own knowledge about animal production.

Argentina has six clearly differentiated regions in terms of population density, economic activities and the socio-economic characteristics of households [12]: the metropolitan area, including surrounding areas of Buenos Aires city (CABA-GBA), the Pampeana region, northwest region, northeast region, Cuyo region and the Patagonia region. The contrasts in lifestyles and cultures of the regions have led to the use of differentiated strategies by the supermarket chains [12].

The objective of the present study was to characterize the Argentinian consumers’ choice behavior toward meat color and fat content in raw beef. For this purpose, the following hypotheses were considered:

-
  **Hypothesis 1.**  *Urban people have a different perception of color and marbling than rural people.*
-
  **Hypothesis 2.**  *Perception of color and marbling, and the purchase habits and beliefs of consumers, depend on consumer socio-demographic characteristics (residence region, age, gender, education level and occupation).*
-
  **Hypothesis 3.**  *Consumer perception of color and marbling depends on purchase habits and beliefs about the importance of the intrinsic cues of meat quality.*
-
  **Hypothesis 4.**  *Consumers can be clustered by their choice behavior and characterized in terms of sociodemographic variables.*

## 2. Materials and Methods

### 2.1. Data Collection

Data were collected through an online survey of people from Argentina, from September to December 2017. The survey was conducted using Google Forms [13] and it consisted of three blocks.

The first block, which described the socio-demographic variables (Table A1), inquired about gender, age range, city and province of residence, profession or occupation, and education level.

In order to describe the lifestyle of the beef consumer (Table A1), the second block asked whether the respondent was the main person responsible for purchasing beef, which venue was their usual place of purchase, and the frequency of beef consumption. 

**Figure A1:**
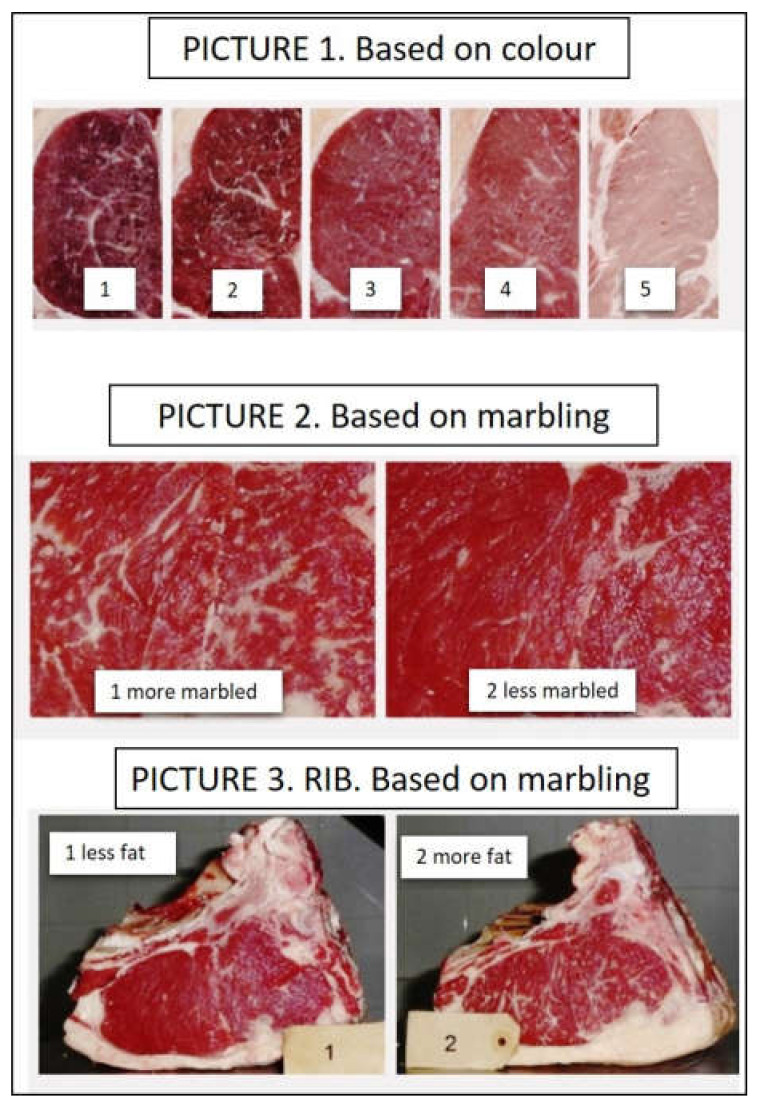
Pictures used in the survey.

The third block, aimed at characterizing consumer preferences in Argentina (Table A1), asked whether the color of muscle and intramuscular fat content were the main characteristics used to describe meat quality. Furthermore, the respondent had to choose one out of five photos of meat (Figure A1, Picture 1) with different colors of muscle and respond to a questionnaire about why they chose it. They could use more than one option to justify their choice: fresh/tender/tasty/juicy/healthy/inexpensive/I do not know/none of the above. Then, the respondent had to choose one of two photos of meat (Figure A1, Picture 2) with different intramuscular fat marbling content and respond a questionnaire about why they chose it. As in the previous question, they could use more than one option to justify their choice: healthy/tender/tasty/juicy/inexpensive/I do not know/none of the above). Finally, the respondent was showed two steak photos (Figure A1, Picture 3) with different levels of fat, were asked to choose one and to justify their choice. Again, they could use more than one option for this: healthy/tender/tasty/juicy/inexpensive/has less waste/has best fat color/adequate intramuscular fat content/has best muscle color/I do not know/none of the above.

Once the survey was available online, the access link was disseminated via e-mail and during a National Animal Farm Show (La Nación Ganadera, 2017). As a result, 1990 surveys were collected.

### 2.2. Statistical Analysis

Data were analyzed using XLSTAT software [14]. Firstly, we calculated a frequency distribution of the sample population according to their socio-demographic characteristics: gender, age, region of residence, education level and occupation. Then, we calculated the frequencies for purchase and consumption habits, for beliefs about meat color and marbling, and for choice behavior. A Kruskal–Wallis test was carried out to test the different hypotheses; that is, the influence of socio-demographic variables or the influence of the purchase habits, or the influence of beliefs on choice behavior. The relationships between a certain effect and the answers of the choice behavior were studied by crosstabs and a chi-square test, with a level of significance of 0.05. To interpret the pattern of association between the studied variables, the adjusted standardized residuals between observed and expected cases in each box were considered at |1.96|.

A decision tree with an exhaustive CHAID analysis method was carried out to search for those consumers with a higher disposition to choose Pictures 1, 2 and 3, respectively. For each decision tree, the choice criteria were the variables in the analysis.

For clustering consumers, three different multifactorial analyses (MFA) were carried out upon the criteria used in each of the pictures to make the selection. We made three different MFA instead of one with all criteria, because possible criteria were not the same for the three different pictures. Criteria with a sum of cosine squared >0.4 were selected to carry out a hierarchical cluster analysis (using Ward’s method for aggregation and Euclidian distance). The cophenetic correlation was calculated as an estimator of the robustness of the clustering. The cophenetic correlation for a cluster tree is defined as the linear correlation coefficient between the cophenetic distances obtained from the tree, and the original distances (or dissimilarities) used to construct the tree. Thus, it is a measure of how faithfully the tree represents the dissimilarities among observations. Finally, a Kruskal–Wallis test was carried out to study the differences between groups of consumers (clusters), and after that, frequencies for socio-demographic variables, purchase habits, beliefs and criteria were calculated to profile the clusters.

## 3. Results and Discussion

### 3.1. Overall Results

Compared with the distribution of population in Argentina according to official statistics (CENSO 2010) [15], the stratification of the sample by region, carried out after obtaining the data, was representative of Argentina. Moreover, the distribution of gender and age within each region (Table 1) was also representative of the country, according to the CENSO 2010 [15].

The general results of the survey are shown in Table 2. Most of the respondents said they were the ones in charge to buy meat in the household (81.3%) and the traditional butcher’s shop was the most common place to buy it (70.2%), a percentage not surprising considering the recent development of the supermarkets in Argentina, especially in rural areas [12]. Beef consumption frequency was bimodal, with alternate days or once a week as the most frequent categories.

Concerning the beliefs, 90% of the respondents agreed with the sentence, “The two main characteristics defining meat quality at purchase time are meat color and marbling”. This is in accordance with Bifaretti [3], in a study focused only on the metropolitan area. In choice behavior (Table 2), options 3 or 4 of Picture 1 (based on color) were chosen most frequently, and “fresh” was the criterion chosen most frequently (67%) to describe Picture 1. This seems to indicate that color is used to infer the freshness of the meat. This is in accordance with Garcia et al. [16] and Verbeke et al. [17], who reported that color is one of the most important fresh beef characteristics at the point of purchase. Consumers related a red-purple color with freshness [18]. Concerning Picture 2 (based on marbling), 86% of the respondents chose the less-marbled steak, and they associated the marbling degree with the “juicy”, “healthy” and “tasty” criteria. In addition, the less fattened rib was chosen more frequently (87%) than the most fattened (Picture 3), and the most frequently marked criteria were “healthy” (52%) and “adequate fat amount” (49%).

### 3.2. Perception of Color and Marbling

#### 3.2.1. Urban Versus Rural Consumers

Worldwide, the urban population is greater than the rural population. In 1950, 30% of the population was urban; in 2014, that value was 54%, and, by 2050, a 66% urban population is projected [19]. In Argentina, as a result of an urbanization process, the rural population decreased rapidly during the twentieth century. In 1999, 13% of the population lived in cities with fewer than 2000 inhabitants (rural population), whereas, by 2010, this percentage decreased to 9% [15]. Urban life is associated with higher literacy and education levels, access to better health systems, and better political/cultural opportunities. However, in the present study, only the frequency of the “fresh” criterion in the question comparing 5 steaks based on color (Table 3; Picture 1) was affected by the place of residence. The percentage of people who chose that criterion for selecting one steak or another was 60%, instead of the expected 56%. Therefore, we can consider it to be a spurious result and dismiss it. In conclusion, regardless of whether the respondents lived in a rural or urban area, they showed similar purchase and consumption habits, beliefs, and choice behavior. Similarly, Zapata et al. [11] found only a slight difference in overall meat consumption between urban and rural consumers; however, they found major differences in the consumption of meat from different animal species, indicating that probably, consumers have a behavior pattern based on which livestock predominates in the region where they live.

#### 3.2.2. Socio-Demographic Characteristics

The present study shows that socio-demographic characteristics influence purchase habits and beliefs, in addition to their effects on the perception of color and marbling.

**Figure 1:**
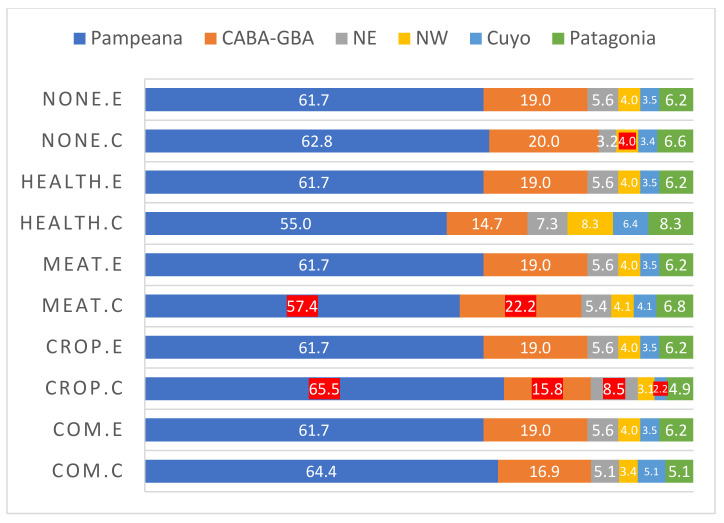
Counted and expected percentages for consumer occupation for each region. CABA-GBA: The metropolitan area, including Greater Buenos Aires. NE: Northeast region. NW: Northwest region. “E”—expected; “C”—counted. None—none of them. Health—human health. Meat—meat production. Crop—crop production. Com.- livestock or meat commercialization. Regions in which counted percentages differed from expected percentages are marked in red.

In general, no differences between socio-demographic characteristics were found between regions for any of the answers, except for occupation (Figure 1). Contrary to the Pampeana region, there are more beef producers and fewer crop producers in the CABA-GBA region than expected. Regarding the criteria used for beef choice, differences between regions were only detected for Picture 3: “juiciness” was chosen more often than expected in the CABA-GBA region (26% instead of 19%) whereas ”better general color” was chosen less often than expected in the northwest region (3% instead of 4%). This result could be related to different animal breeds farmed in the different regions, which produce different beef qualities. Angus and Hereford are the main breeds that supply the beef market in the CABA-GBA region, whereas Criollo, Bradford and Brangus are the main breeds farmed in the northwest region. As for Hypothesis 1, these findings were considered spurious, without practical relevance. In conclusion, consumer perception of color and marbling is not dependent on the region where the respondents live.

Education level did not influence purchase habits, beliefs, or choice behavior, but consumer occupation influenced the frequency of beef consumption and choice behavior. Gender only affected the belief question; 87% of men agreed with the idea that color and marbling are the main attributes at purchase time, whereas 92.2% of women agreed with that statement. Several studies [20,21,22] reported differences between men and women in terms of meat consumer perception. For instance, modern Italian consumers are worried about animal welfare, with women more sensitive to it; they perceive this attribute more strongly than men do as indicative of meat quality [23]. Similar results were found by Schnettler et al. [24] in the Chilean population: women had different animal welfare expectations and they wanted more information about animal welfare than men.

**Figure 2:**
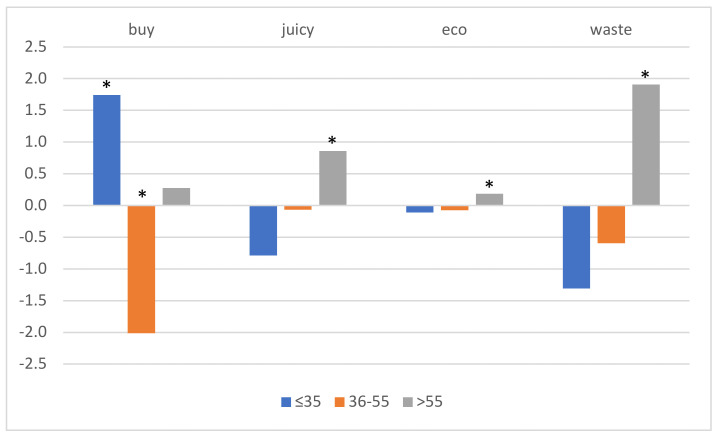
Expected minus counted percentages for variables and criteria affected by consumer age. * Groups in which differences were significant are marked with an asterisk; -buy-, “Are you the person in charge of beef-buying at home?”; -juicy- and –eco-, based on the color of the five steaks; -waste-, chosen rib.

Concerning consumer age, differences found in purchase habits and in choice behavior are represented in Figure 1. The youngest consumers (≤35 years old) were in charge of buying beef less frequently than expected, whereas the contrary happened for people 36–55 years old. However, the three criteria that were affected by age were only chosen by people >55 years old (Figure 2; Table 4).

Since choice behavior and belief depended on gender, age and occupation, and purchase habits depended on age, hypothesis 2 should not be rejected.

As consumer gender, age and occupation influenced purchase habits and choice behavior, they can be considered as a consumer clustering.

#### 3.2.3. Purchase Habits and Belief

Being or not being the person in charge of buying beef in the household did not influence the consumer’s choice behavior but did influence their beliefs (Table 5). People in charge of buying the beef agreed with the sentence about color and marbling slightly more frequently than expected (90.7% vs. 89.9%), whereas the agreement was slightly less frequent than expected (86.6% vs. 89.9%) for people not in charge of buying the meat. Nevertheless, most people, independently of being in charge or not in charge, agreed with the idea that color and marbling are relevant at purchase time (Table 5), supporting the conclusions of Bifaretti [2]. Many other studies have demonstrated that consumers use a visual appraisal to infer sensory quality [25,26,27,28].

Beef consumption frequency affected the choice and some of the criteria used to select options of Picture 1 and Picture 2 (Table 5). In addition, differences were found for the question about the importance of color and marbling at the moment of purchase. Only 75% of the people eating beef once a month agreed with the sentence, whereas the main average was 87%. The previous experience with the product and the frequency of consumption as factors influencing consumer perception of a certain food product were already stated by several authors [25,29,30].

The degree of agreement with the sentence about color and marbling importance influenced the choice of Picture 1 as well as some criteria used in the choices of Picture 2 and Picture 3. Surprisingly, an “adequate fat amount” of the rib was marked as important more frequently for people in disagreement with the sentence (56%) than for the people in agreement with it (48%), and “better general color” was not affected by the belief about the importance of color and marbling.

#### 3.2.4. Decision Trees for Choices on Pictures as a Function of Significant Socio-Demographic Variables, Purchase Habits and Beliefs

It is well known that both the place of residence and socio-economic context have an influence on choice and pattern behavior [31]. Moreover, ethics, religious beliefs and traditions influence beef consumption [32]. In addition, consumer perception can be influenced by attitudes and beliefs about the characteristics of certain products and the way they are produced, handled, or distributed.

**Figure 3:**
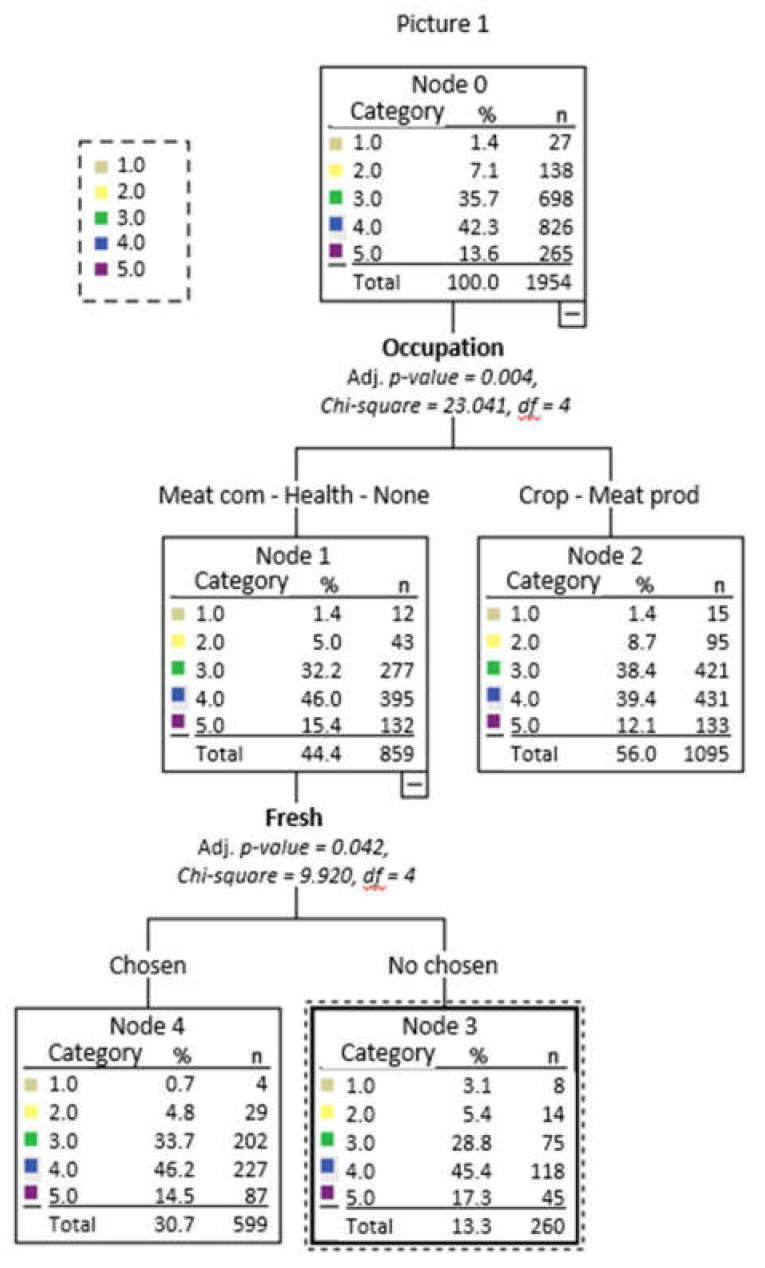
Decision tree for Picture 1 as a function of sociodemographic variables, purchase habits, beliefs and the criteria used in the choice.

**Figure 4:**
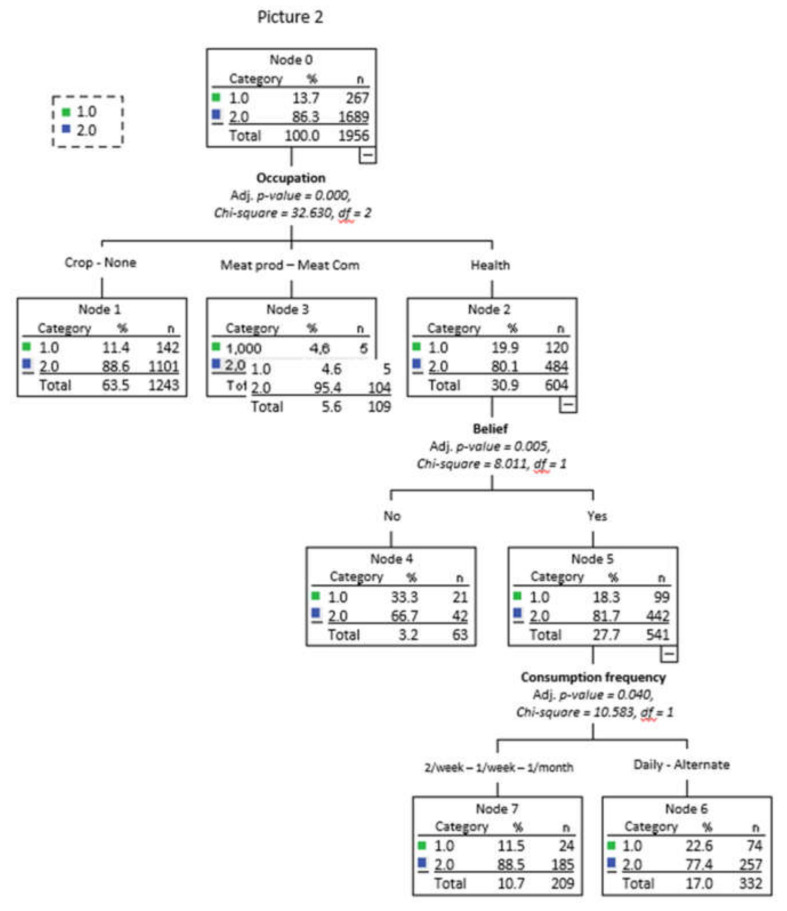
Decision tree for Picture 2 as a function of socio-demographic variables, purchase habits, beliefs and criteria used in the choice.

**Figure 5:**
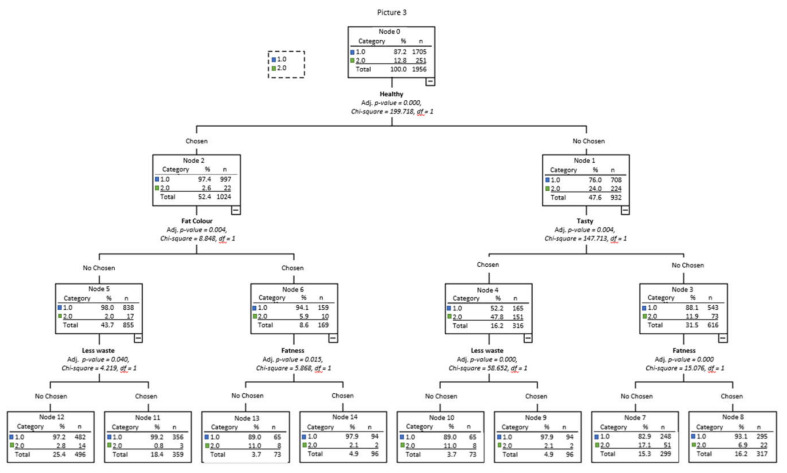
Decision tree for Picture 3 as a function of sociodemographic variables, purchase habits, beliefs, and the criteria used in the choice.

Figure 3, Figure 4 and Figure 5, respectively, show the decision trees for the choices of Pictures 1, 2, and 3 as a function of the socio-demographic variables, purchase habits, and beliefs that were significant (that is, gender, age, activity, place of purchase, frequency of beef consumption, and belief about color and marbling importance), as well as on the criteria used for choosing each of the pictures.

For Picture 1, 100% of the respondents were correctly forecasted as selecting option 4 of the picture and their occupation, and freshness seemed to be the most important criterion (Figure 3). It is well known [16,17,33] that at the point of purchase, the color of fresh beef is one of the most important characteristics to the consumer.

The tree for Picture 2 (Figure 4) correctly classified 100% of the respondents in option 2 (less marbling). The percentage of the respondents selecting option 2 was higher when their occupation was related to human health (node 3, 95.4%). In this sense, the fact that consumers chose a lean option is in accordance with the recommendation of the World Health Organization [34]. Node 2 grouped people who work on occupations related to meat production or commercialization and separated them into two nodes as a function of their beliefs. People who agreed with the importance of color and marbling (node 4, 81.7%) chose option 2 of the pictures more often than did people in disagreement with it (node 5, 66.7%). In this sense, marbling is considered an important beef quality trait throughout the world because it is associated with a positive eating experience [35], but, contrary to what is thought, most workers in the beef industry chose the lean option. Finally, node 4 was divided into two groups based on their beef consumption frequency. At a higher frequency of consumption, a lower percentage of people chose option 2 of Picture 2.

In the tree for Picture 3 (Figure 5), 89.3% of the answers were correctly forecasted. Eighty-seven percent of the respondents chose rib 1 (less fat). This percentage raised to 97% in node 1, which comprised people who chose “healthy” as a criterion to select the less fattened rib. This node 1 was divided into two as a function of the fat color criterion. People who did not mark the “fat color” criterion (node 3) were divided according to the “less waste” criterion. Node 4 was also divided into two as a function of the “adequate marbling” criterion. On the other side, node 2 represented people that did not mark the “healthy” criterion as important. In this node, rib 1 was chosen less frequently than in node 1 (76%). Node 2 was divided into two as a function of the *tasty* criterion. If “tasty” was unmarked (node 6), rib 1 was chosen by 88% of the respondents, whereas, if “tasty” was marked as important (node 5), only 52% chose rib 1. Node 5 was divided into two groups depending on the “less waste” criterion; people marking it as important chose rib 1 (node 12), whereas people who did not mark it (node 11) chose rib 2. It is the only node in which rib 2 was more frequently chosen than rib 1. Finally, node 6 was divided into two depending on “adequate marbling”; rib 1 was more frequently selected when the marbling criterion was marked (node 14) than when it was unmarked (node 13). In general terms, the highest frequency of the choice of rib 1 was in node 8 (“healthy”, “less waste”, “fat color” not important) and the highest frequency of choice of rib 2 was in node 11 (“tasty”, “healthy” not important, “less waste” not important).

Clearly, there are two target markets in Argentina based on beef fat levels: one for people who are interested in their health and want lean beef with an adequate color, and another market for people who want palatability. Respondents associated the degree of marbling with “juicy”, “healthy”, and “tasty” criteria. They associated the less fattened rib with “healthy and adequate amount of fat”. Differences in fat preferences have been found between geographical regions [36]; for instance, slightly visible fat in beef (including cover fat and intramuscular fat) was preferred in some countries such as Spain [37,38].

#### 3.2.5. Multiple Factor Analysis

Table 6 shows the percentage of variability explained by the two first factors for each of the multiple factor analyses (MAF) carried out, as well as the cosine squared for each variable in each factor.

In the MAF of Picture 1, the first two factors explained 41.9% of the variability. “Tender”, “tasty” and “juicy” criteria presented a sum of cosine squared > 0.4 and were therefore selected for the hierarchical cluster. In the MAF of Picture 2, 68% of the variability was explained by the first two factors and the selected criteria were “tender”, “tasty”, “juicy” and “healthy”. In the MAF of Picture 3, 36.1% of the variability was explained by the first two factors and selected criteria were “tender”, “tasty”, “juicy”, “fat color”, and “general color”.

Three groups of consumers were obtained from the cluster analysis, with a cophenetic correlation of 0.456. The description of consumer profiles (clusters) according to their socio-demographic variables, purchase habits and beliefs, and by their choice behavior, are shown in Table 7; Table 8 respectively.

No differences between groups were found for consumer gender, consumer age, or beef consumption frequency (*p* > 0.05), but occupation differed between consumer groups (*p* < 0.001). In the same way, no differences were found between groups in the chosen Picture 1 or chosen Picture 2 categories (*p* > 0.05), but differences were found for the chosen Picture 3 category (*p* < 0.001) between the three different groups.

The first cluster (*n* = 751, 38.3% of the sample) comprises respondents who showed a profile that could be termed as “hedonic”. To choose the pictures, they used the criteria “tender”, “tasty” and “juicy”, whereas “healthy” or “color” was less frequently chosen than expected. A greater proportion of them preferred the second option of Picture 3; that is, the most fattened. According to Smith and Carpenter [39], tenderness, flavor, and juiciness are the primary traits to describe overall beef palatability. Moreover, according to Lusk et al. [40], these primary traits are highly correlated with overall experienced quality, intention to purchase, and willingness to pay. Thus, this group is characterized by choosing based on palatability. In this group, we found the most people whose occupation was related to crop production (33.8%). The second group (n = 734, 37.4% of the sample) selected the criterion “healthy” in Picture 2 and in Picture 3, but they did not mark any of the other criteria as important and they cannot be defined in terms of occupation. Thus, they could be classified as “health-conscious”. They chose the less fattened Picture 3 as recommended by the WHO [34] to decrease the number of calories in their meals. The third group (*n* = 475, 24.2%) chose “fresh” and “healthy” for Picture 1, no particular criteria for Picture 2 and “less waste”, “better fat color”, and “better general color” for Picture 3; that is, they were people that use general appearance to choose the pictures. Visual appearance characteristics are highly related to consumer expectations and are intrinsic quality cues [17]. Moreover, because these characteristics are used to access food quality, they are highly related to their choice at purchase [41]. Consumers from the third group were not worried about tenderness, juiciness, taste, or health, although, curiously, they were mostly occupied in human health-related jobs. Although clusters could not be defined in terms of consumers’ age, people in the “appearance” group tended to be the youngest (≤35 years old); this could explain their lack of concern with the “healthy” criterion.

Consumers are the last link of the production chain, and they have their own expectations about the product, associated with their beliefs and/or feelings. According to Deliza et al. [42], previous information and experiences form the expectation process. In this sense, the frequency of consumption influences the expectation process; indeed, it influences the perception of beef quality, as shown in the present study. Since there is little information about fresh meat, consumers have difficulties in forming their quality expectations. According to Grunert et al. [43], labeling and appearance are the main characteristics that form meat quality expectations. However, they do not seem to be very good predictors of meat-eating quality.

The three groups of consumers identified in Argentina are important for marketing strategies, as they have their own characteristics. While consumers in the “hedonic” group search for a pleasurable sensory experience, consumers in the “appearance” group search for visual aspects, and those in the “health-conscious” group are interested in a healthy diet.

## 4. Conclusions

In order to generate a beef marketing strategy in Argentina, it was possible to group the population into three market groups, named “health-conscious”, “hedonic” and “appearance”. The first group chooses lean beef because it is healthier. In turn, the second group prefers fattier beef, associating it with a tender, tasty and juicy steak, looking for palatability. Consumers in the third group make their choice based on how beef looks like and how it relates to freshness, color, health and the lower production of waste (less waste). On the other hand, the decision tree grouped the Argentine population into two market groups based on beef fat content. The first group includes the “health-conscious” and “appearance” groups, and it contains consumers interested in their health (lean meat) and in a given beef color. The other group contains the “hedonic” group, which consists of consumers who search for a palatable product. Fat and color in beef are the main attributes that all groups have in common and consumer’s beliefs and purchase habits are influenced by them. As beliefs and purchase habits appear to be influenced by socio-demographic characteristics, we could consider that the consumer perception of color and marbling depends on these.

## Figures and Tables

**Table 1:** Socio-demographic characteristics of the respondents. Data are percentages of valid answers, shown for each of the administrative regions of Argentina.

		Pampeana ^1^	CABA-GBA ^2^	North East	North West	Cuyo	Patagonia
Gender	Male	65.2	66.0	66.4	58.2	64.2	61.7
	Female	34.8	34.0	33.6	41.8	35.8	38.3
Age	≤35	42.2	46.6	39.1	36.7	44.8	45.8
	36–55	36.5	34.3	44.5	39.2	43.3	40.8
	>55	21.2	19.0	16.4	24.1	11.9	13.3
Highest education level reached	Primary school	1.9	0.3	2.7	1.3	None	0.8
	Secondary school	24.4	25.9	16.4	20.3	25.4	22.5
	Tertiary or higher	73.7	73.9	80.9	78.5	74.6	76.7
Occupation	Crop production	30.3	23.8	43.5	22.1	17.9	22.5
	Meat production	24.8	31.1	25.9	27.3	31.3	29.2
	Livestock or meat commercialization	3.2	2.7	2.8	2.6	4.5	2.5
	Human health	5.0	4.4	7.4	11.7	10.4	7.5
	None of the above	36.7	38.0	20.4	36.4	35.8	38.3

^1^ except CABA-GBA. ^2^ Metropolitan area (Buenos Aires city and conurbation (Greater Buenos Aires)).

**Table 2:** Survey questions and answers of purchase habits, beliefs, and choice behavior. Results are percentages of valid answers.

Question	Response		%
**Purchase and consumption habits**			
Are you the person in charge of beef-buying at home?	Yes		81.3
	No		18.7
Where do you buy beef most often?	At the supermarket, packaged		11.5
	At the supermarket, butcher’s		18.3
	Traditional butcher’s shop		70.2
How often do you eat beef?	Daily		16.7
	Alternate days		38.3
	Twice a week		12.3
	Once a week		29.6
	Once a month		3.1
**Beliefs**			
Do you agree with the following sentence: “The two main characteristics defining beef quality at purchase time are beef color and marbling”	Yes		89.9
	No		10.1
**Choice behaviour**			
Based on the color of the following five steaks, which one would you choose? (Picture 1)	Option 1 (darker)		1.4
	Option 2		7.0
	Option 3		35.6
	Option 4		42.1
	Option 5 (lighter)		13.5
	Justification of choice ^1^	Fresh	67.0
		Tender	42.9
		Tasty	36.5
		Juicy	14.3
		Healthy	31.3
		Cheap	0.6
		None of the above	2.1
Based on the marbling of the following two steaks, which one would you choose? (Picture 2)	Option 1 (more marbling)		13.6
	Option 2 (less marbling)		86.2
	Justification of choice ^1^	Tender	26.9
		Tasty	65.2
		Juicy	87.6
		Healthy	75.6
		Cheap	0.0
		None of the above	2.3
In general, which of the following two ribs would you choose? (Picture 3)	Option 1 (less fattened)		87.2
	Option 2 (more fattened)		12.8
	Justification of choice ^1^	Tender	23.7
		Tasty	31.3
		Juicy	11.9
		Healthy	52.3
		Cheap	0.1
		Less waste	38.6
		Better fat color	18.6
		Adequate fat amount	49.1
		Better general color	34.3
		None of the above	1.1

^1^ Percentage of respondents that used each criterion.

**Table 3:** Chi-square *p*-values for the Kruskal–Wallis test, with urban/rural or region criteria as the main effect.

Description	Urban/Rural	Region
Gender	<0.001	0.785
Age	0.654	0.286
Education level	0.083	0.615
Your occupation is related to…	<0.001	0.003
Are you the person in charge of beef buying at home?	0.106	0.119
Where do you buy beef most often?	0.263	0.115
How often do you eat beef?	0.789	0.368
The two main characteristics… are beef color and marbling	0.937	0.292
Based on the color of the five steaks	0.324	0.306
Fresh	0.017	0.197
Tender	0.511	0.284
Tasty	0.285	0.592
Juicy	0.867	0.419
Healthy	0.226	0.838
Cheap	0.191	0.597
None of the above	0.728	0.401
Based on the marbling of the two steaks	0.738	0.686
Tender	0.092	0.862
Tasty	0.585	0.853
Juicy	0.541	0.467
Healthy	0.303	0.607
Cheap	1.000	1.000
None of the above	0.843	0.996
Chosen rib	0.356	0.841
Tender	0.675	0.886
Tasty	0.604	0.153
Juicy	0.648	0.044
Healthy	0.563	0.938
Cheap	0.775	0.987
Less waste	0.078	0.112
Better fat color	0.484	0.156
Adequate fat amount	0.886	0.856
Better general color	0.507	0.050
None of the above	0.058	0.563

**Table 4:** Chi-square *p*-values for the Kruskal–Wallis test with socio-demographic variables (gender, age, education level and occupation) as the main effects.

	Gender	Age	Education	Occupation
Are you the person in charge of beef buying at home?	0.685	>0.001	0.102	0.074
Where do you buy beef most often?	0.226	0.419	0.951	0.378
How often do you eat beef?	0.241	0.982	0.860	0.000
The two main characteristics… are beef color and marbling	0.014	0.930	0.780	0.089
Based on the color of the five steaks	0.282	0.588	0.611	0.001
Fresh	0.096	0.100	0.851	0.000
Tender	0.682	0.822	0.131	0.371
Tasty	0.645	0.645	0.323	<0.001
Juicy	0.371	0.047	0.486	0.472
Healthy	0.932	0.630	0.720	0.082
Cheap	0.895	0.031	0.897	0.309
None of the above	0.891	0.435	0.133	0.086
Based on the marbling of the two steaks	0.122	0.514	0.561	<0.001
Tender	0.864	0.606	0.490	0.243
Tasty	0.377	0.265	0.674	<0.001
Juicy	0.408	0.673	0.319	0.260
Healthy	0.611	0.166	0.832	<0.001
Cheap	1.000	1.000	1.000	1.000
None of the above	0.566	0.376	0.661	0.522
Chosen rib	0.563	0.596	0.856	0.122
Tender	0.952	0.454	0.866	0.924
Tasty	0.720	0.986	0.322	0.001
Juicy	0.651	0.275	0.310	0.314
Healthy	0.550	0.443	0.177	0.018
Cheap	0.174	0.430	0.250	0.002
Less waste	0.864	0.027	0.215	0.452
Better fat color	0.711	0.391	0.837	0.645
Adequate fat amount	0.739	0.599	0.690	0.034
Better general color	0.721	0.422	0.863	0.007
None of the above	0.309	0.317	0.601	0.863

**Table 5:** Chi-square *p*-values for the Kruskal–Wallis test with purchase habits and beliefs as main effects.

Description	Are You the Person in Charge of Beef Buying at Home?	Where Do You Buy Beef More Often?	How Often Do You Eat Beef?	The Two Main Characteristics Are Beef Color and Marbling
Based on the color of the five steaks	0.747	0.409	0.040	0.021
Fresh	0.133	0.724	0.267	0.241
Tender	0.987	0.653	0.436	0.863
Tasty	0.971	0.258	0.001	0.378
Juicy	0.906	0.702	0.909	0.221
Healthy	0.966	0.672	0.971	0.875
Cheap	0.573	0.237	0.017	0.244
None of the above	0.586	0.964	0.820	0.550
Based on the marbling of the two steaks	0.534	0.376	<0.001	0.022
Tender	0.136	0.171	0.131	0.284
Tasty	0.859	0.697	0.976	0.084
Juicy	0.184	0.158	0.332	0.005
Healthy	0.731	0.450	0.711	0.018
Cheap	1.000	1.000	1.000	1.000
None of the above	0.876	0.662	0.144	0.785
Chosen rib	0.399	0.024	0.690	0.120
Tender	0.910	0.365	0.638	0.996
Tasty	0.347	0.132	0.359	0.510
Juicy	0.790	0.037	0.323	0.423
Healthy	0.609	0.068	0.848	0.595
Cheap	0.632	0.108	0.807	0.737
Less waste	0.796	0.498	0.634	0.604
Better fat color	0.762	0.157	0.124	0.230
Adequate fat amount	0.713	0.904	0.156	0.027
Better general color	0.871	0.430	0.203	0.876
None of the above	0.246	0.560	0.805	0.874
The two main characteristics are beef color and marbling	0.012	0.923	<0.001	

**Table 6:** Eigenvalue and percentage of variability explained by the first two factors for each of the three Multiple Factor Analysis carried out, and cosine squared for each variable in each factor. The criterion “none of the above” was excluded from the analysis. When the sum of the cosine squared in the two factors was >0.4, the criterion was chosen for the hierarchical cluster (values in bold).

		Factor 1	Factor 2
Picture 1 (based on color)	Eigenvalue	1.141	1.004
	Variability (%)	22.286	19.606
	Cosine squared		
	*Fresh*	**0.056**	**0.650**
	*Tender*	**0.335**	**0.186**
	*Tasty*	**0.488**	**0.110**
	*Juicy*	**0.395**	**0.006**
	*Healthy*	0.077	0.192
	*Cheap*	0.004	0.000
Picture 2 (based on marbling)	Eigenvalue	1.489	0.583
	Variability (%)	48.854	19.116
	Cosine squared		
	*Tender*	**0.441**	**0.307**
	*Tasty*	**0.515**	**0.214**
	*Juicy*	**0.464**	**0.112**
	*Healthy*	**0.533**	**0.131**
	*Cheap*	0.008	0.002
Picture 3 (Rib) (based on marbling)	Eigenvalue	1.415	1.052
	Variability (%)	20.699	15.382
	Cosine squared		
	*Tender*	**0.436**	**0.016**
	*Tasty*	**0.484**	**0.000**
	*Juicy*	**0.611**	**0.015**
	*Healthy*	0.031	0.001
	*Cheap*	0.029	0.009
	*Less waste*	0.099	0.252
	*Fat color*	**0.004**	**0.575**
	*Fat amount*	0.017	0.046
	*General color*	**0.000**	**0.687**

**Table 7:** Percentages of each socio-demographic characteristic, purchase habits, and beliefs for each of the consumer groups obtained in the cluster analysis.

		Consumer Group			
Description		Hedonic (38.3%)	Health-Conscious (37.4%)	Appearance (24.2%)	*p*-Value
Gender	Male	65.6 *	64.4	64.2	0.841
	Female	34.4	35.6	35.8	
Age	≤35	41.1	42.4	46.7	0.195
	36–55	38.1	38.6	34.5	
	>55	20.8	19.6	18.7	
Highest education level reached	Primary school	1.2	1.5	1.9	0.272
	Secondary school	26.1	23.7	21.1	
	Tertiary or higher	72.7	74.8	77.0	
Occupation	Crop production	33.8	27.2	*22.2*	0.003
	Meat production	*22.8*	28.9	29.3	
	Livestock or meat commercialization	37.0	3.0	2.1	
	Human health	4.9	4.75	8.3	
	None of the above	34.9	36.2	38.0	
Are you the person in charge of beef buying at home?	Yes	80.6	82.3	81.1	0.683
	No	19.4	17.7	18.9	
Where do you buy beef most often?	At the supermarket, packaged	11.7	11.7	10.6	0.221
	Butcher’s at the supermarket	20.3	17.6	16.4	
	Traditional butcher’s shop	68.0	70.6	73.0	
How often do you eat beef?	Daily	15.7	16.8	18.2	0.221
	Alternate days	37.7	38.7	38.7	
	Twice a week	11.8	12.7	12.4	
	Once a week	30.7	29.4	28.0	
	Once a month	4.2	2.3	2.8	
Do you agree with the following sentence: “The two main characteristics defining beef quality at purchase time are beef color and marbling”	Yes	88.9	91.1	89.5	0.351
	No	11.1	8.9	10.5	

* Percentages higher than expected are marked in bold, and those lower than expected are marked in italics.

**Table 8:** Description of consumer profiles (clusters) according to their choice behavior. Percentages are of people who marked a criterion as used in the choice of each picture.

Description		Hedonic (38.3%)	Health- Conscious (37.4%)	Appearance (24.2%)	*p*
Based on the color of the following five steaks, which one would you choose? (Picture 1)	Option 1 (darker)	1.5	1.6	0.8	0.923
	Option 2	7.4	6.1	8.0	
	Option 3	35.6	36.3	35.1	
	Option 4	42.5	42.6	41.4	
	Option 5 (lighter)	13.1	13.4	14.6	
	Fresh ^1^	62.3	63.6	79.6	<0.001
	Tender	49.0	36.1	43.6	<0.001
	Tasty	56.2	22.2	27.6	<0.001
	Juicy	24.4	9.7	5.5	<0.001
	Healthy	26.1	31.2	39.8	<0.001
	Cheap	0.8	0.3	0.8	0.327
	None of the above	2.9	1.5	1.7	0.001
Based on the marbling of the following two steaks, which one would you choose? (Picture 2)	Option 1 (more marbling)	13.2	14.9	12.4	0.452
	Option 2 (less marbling)	86.8	85.1	87.6	
	Tender	37.8	15.4	27.6	<0.001
	Tasty	72.3	3.7	23.8	<0.001
	Juicy	28.0	1.9	4.2	<0.001
	Healthy	56.1	94.6	23.8	<0.001
	Cheap	0.0	0.0	0.0	1.000
	None of the above	3.3	0.7	3.2	0.001
In general, which of the following two ribs would you choose? (Picture 3)	Option 1 (less fattened)	77.8	95.4	89.3	<0.001
	Option 2 (more fattened)	22.2	4.6	10.7	
	Tender	36.6	13.5	19.2	<0.001
	Tasty	72.6	1.2	12.4	<0.001
	Juicy	26.6	3.1	2.1	<0.001
	Healthy	45.8	62.1	47.4	<0.001
	Cheap	0.1	0.0	0.0	0.447
	Less waste	36.5	37.7	43.2	0.055
	Better fat color	9.2	0.5	61.3	<0.001
	Adequate fat amount	48.7	48.9	50.1	0.886
	Better general color	24.4	2.0	100	<0.001
	None of the above	1.6	1.4	0.0	0.026

^1^ For each choice criterion, the table only shows the percentage of times in which a certain criterion was chosen. Percentages higher than expected are marked in bold and those lower than expected are marked in italics.

## Data Availability

The data from presented in this study are available on request from the corresponding author. Although consumer data have been anonymised, data are not publicly available.

## Appendix A

**Table A1:** Questionnaire on consumer socio-demographic characteristics, purchase and consumption habits, beliefs and choice behavior.

Socio-Demographic Variables	
Gender ^1^	
Age ^2^	
Education level ^3^	
Region of residence ^4^/City ^5^	
Your occupation is related to ^6^	
Purchase and consumption habits	
Are you the person in charge of beef-buying at home? ^7^	
Where do you buy beef more often? ^8^	
How often do you eat beef? ^9^	
Beliefs	
Do you agree with the following sentence: “The two main characteristics defining beef quality at purchase time are beef color and marbling”. ^1^	
Choice behavior	
Based on the color of the following five steaks, which one would you choose? ^10^	
Why? I consider that the chosen steak is more…	
Fresh	
Tender	
Tasty	
Juicy	
Healthy	
Cheap	
None of the above	
Based on the marbling of the following two steaks, which one would you choose? ^10^	
Why? I consider that the chosen steak is more…	
Tender	
Tasty	
Juicy	
Healthy	
Cheap	
None of the above	
In general, which of the following two ribs would you choose? ^10^	
Why? I consider that the chosen steak is more… ^11^	
Tender	
Tasty	
Juicy	
Healthy	
Cheap	
Less waste	
Better fat color	
Adequate fat amount	
Better general color	
None of the above	

^1^ Male, female. ^2^ <35, 36–55, >56 years old. ^3^ Primary school, secondary school, tertiary or higher. ^4^ Pampeana, CABA-GBA, North East, North West, Cuyo, Patagonia. ^5^ Urban, rural. ^6^ Crop production, meat production, livestock or meat commercialization, human health, none of the above. ^7^ True or false response. ^8^ At the supermarket, packaged; butcher’s at the supermarket; traditional butcher’s shop. ^9^ Daily; alternate days, twice a week, once a week, once a month. ^10^ Check only one. ^11^ You can check various options.
